# Microbial Exchange via Fomites and Implications for Human Health

**DOI:** 10.1007/s40726-019-00123-6

**Published:** 2019-08-31

**Authors:** Brent Stephens, Parham Azimi, Megan S. Thoemmes, Mohammad Heidarinejad, Joseph G. Allen, Jack A. Gilbert

**Affiliations:** 1 Department of Civil, Architectural, and Environmental Engineering, Illinois Institute of Technology, Alumni Memorial Hall 228E, 3201 South Dearborn Street, Chicago, IL 60616 USA; 2 Environmental Health Department, Harvard T.H. Chan School of Public Health, Boston, MA USA; 3 Department of Pediatrics, University of California San Diego School of Medicine, San Diego, CA USA

**Keywords:** Microbiology, Built environment, Contamination, Infectious disease transmission, Aerosol, Quantitative microbial risk assessment (QMRA)

## Abstract

**Purpose of Review:**

Fomites are inanimate objects that become colonized with microbes and serve as potential intermediaries for transmission to/from humans. This review summarizes recent literature on fomite contamination and microbial survival in the built environment, transmission between fomites and humans, and implications for human health.

**Recent Findings:**

Applications of molecular sequencing techniques to analyze microbial samples have increased our understanding of the microbial diversity that exists in the built environment. This growing body of research has established that microbial communities on surfaces include substantial diversity, with considerable dynamics. While many microbial taxa likely die or lay dormant, some organisms survive, including those that are potentially beneficial, benign, or pathogenic. Surface characteristics also influence microbial survival and rates of transfer to and from humans. Recent research has combined experimental data, mechanistic modeling, and epidemiological approaches to shed light on the likely contributors to microbial exchange between fomites and humans and their contributions to adverse (and even potentially beneficial) human health outcomes.

**Summary:**

In addition to concerns for fomite transmission of potential pathogens, new analytical tools have uncovered other microbial matters that can be transmitted indirectly via fomites, including entire microbial communities and antibiotic-resistant bacteria. Mathematical models and epidemiological approaches can provide insight on human health implications. However, both are subject to limitations associated with study design, and there is a need to better understand appropriate input model parameters. Fomites remain an important mechanism of transmission of many microbes, along with direct contact and short- and long-range aerosols.

## Introduction

Conceptualized as early as the 1500s, fomites (or *fomes*) were first thought of as “seeds of disease,” found in the clothing of infected individuals that spread contagion long distances by indirect human contact [1]. Today, fomites are generally considered any inanimate object that, when contaminated with infectious organisms, can serve as a means of transferring disease-causing agents to a new human host. Because people in industrialized countries spend approximately 90% of their time indoors [2], the most important fomites for contimination and transmission tend to be those found in the built environment and those that humans frequently come into direct contact with, such as doorknobs, countertops, medical equipment, handrails, clothing, and mobile phones. As our understanding of microbes in the built environment has greatly expanded in the last decade, so has our understanding of fomites and their role in the transmission of infectious agents and other microbial matter to and from humans. Here, we review the recent body of literature on fomite contamination and microbial survival in the built environment, factors that affect transmission of microbes between fomites and humans, and the implications for human health. Table 1 summarizes what the authors consider to be some of the most important or influential recent research outcomes within the major categories of articles reviewed herein. These highlighted articles were determined to stand out in the field as being particularly novel, comprehensive, and/or addressing a fundamental question that had not been thoroughly addressed before.

**Table 1:** Summary of recent research on microbial exchange via fomites and implications for human health

Subject area	Reference(s)	Key research outcomes
Microbes on surfaces		
Microbial communities on fomite surfaces		
Indoor microbiomes	Lax et al. [3••] Chase et al. [4••]	Humans deposit their own microbial signatures on indoor surfaces, but microbial communities are generally considered to remain inactive or dormant until being transferred to other host locations or experiencing an influx of nutrients.
Viral pathogens on fomite surfaces		
Presence and abundance	Stobnicka et al. [5•]	Viruses that are known to cause communicable diseases in humans are quite commonly found on surfaces in the built environment.
Viability and survival	Thompson and Bennett [6•]	Many viral pathogens survive and remain active on fomite surfaces over time, even for several days, influenced by a combination of material type, environmental conditions, virus strain, inoculation methods, and viral detection methods. Moreover, viral pathogens usually survive longer on non-porous materials than on porous materials.
Bacterial hazards on fomite surfaces		
Potentially pathogenic bacteria	Malcolm et al. [7•]	Potentially pathogenic bacteria, such as Mycobacterium abscessus, deposit and remain viable on fomite surfaces in the built environment.
Antibiotic-resistant bacteria	Missri et al. [8•] Smibert et al. [9••] Haun et al. [10•] Jackson et al. [11••] Hartmann et al. [12••] Mahnert et al. [13••]	Antibiotic-resistant bacteria deposit and remain on a wide variety of fomite surfaces (e.g., mobile phones, keyboards, and clothing), particularly in healthcare settings. However, abundance of antibiotic-resistant bacteria is often low, suggesting fomite transmission is possible but often unlikely. Moreover, the use of antimicrobial chemical cleaners can adversely impact microbial communities on surfaces and in surface dust by increasing the abundance of antibiotic resistance genes (ARGs).
Transmission between fomites and humans		
Measurements of microbial transfer to/from fomites		
Surface-scale	Greene et al. [14•]	Microbes can physically transfer between fomite surfaces and humans via touching, but the transmission efficiency depends on the surface material, hand coverings, material hydrophobicity, and moisture content of contact surfaces.
Room-scale	Killingley et al. [15•] Kunkel et al. [16•] Reynolds et al. [17••]	Room-scale experiments have demonstrated the importance of fomites in the transmission of microbes between humans and surfaces in the built environment. Bioaerosols can contaminate surfaces up to several meters away from the source. Bacterial tracer studies have been used recently to confirm fomites as a key transmission pathway.
Mathematical modeling of fomite transmission		
Mechanistic models	Xiao et al. [18•]	Mechanistic quantitative microbial risk assessment (QMRA) models can elucidate the likely dominant transmission pathways for microbial hazards by integrating a variety of model inputs.
Improving model inputs	Zhang and Li [19•] Greene et al. [20•] Weir et al. [21•]	QMRA models require accurate inputs to produce accurate outputs. Recent studies have incorporated improved model inputs such as detailed human activity patterns, microbial transfer efficiencies, and microbiological sampling recovery.
Epidemiology of fomite transmission		
Microbial pathogens	Kutter et al. [22•]	Epidemiological investigations offer the benefit of increasing understanding of overall disease transmission and attack rates in exposed populations, but are often limited in their ability to disentangle the role of various exposure routes.
Indoor microbiome	Dannemiller et al. [23••] O’Connor et al. [24•]	Increased microbial diversity and abundance of certain taxa on home surfaces early in life, which are shaped by occupancy, maintenance, and building characteristics, are associated with decreased asthma risk in epidemiology studies, suggesting microbial exposures can shape our innate immune responses to protect against allergy and asthma.

## Indoor Microbiomes

We live in a microbial world. Viruses, bacteria, protists, fungi, and archaea exist in all of our inhabited environments [25–27]. In buildings, we shed microbes directly to the indoor air and onto building surfaces [28–30], microbes are transported indoors from outdoors [31, 32], and we also acquire microbes from our surroundings [3••, 33, 34]. Human occupancy and activity, the outdoor environment, and building design and operation each influence the abundance and diversity of microbes in buildings—or what is collectively referred to as the *indoor microbiome* [23••, 35–42]. Many molecular analyses have identified considerable microbial diversity on built surfaces. Most microbes found in indoor environments appear to be dormant, inactive, or dead [43], and either has no known impact on human health or are possibly even beneficial to human health [24•, 44–46]. For example, early life exposures to particular microbes or assemblages of microbes have been shown to shape our innate immune responses to protect against allergy and asthma [47–49]. However, potentially pathogenic organisms can also reside within the microbial milieu of our built worlds, which can have a variety of negative health consequences.

## Microbes on Surfaces

Inanimate objects in the built environment can serve as reservoirs of microbial matter. Each of these objects is host to an entire community composed of a wide variety of bacterial, viral, archaeal, protistan, and fungal organisms, including potential pathogens and microbial metabolic products harmful to humans.

### Microbial Community Ecology on Fomite Surfaces

On indoor surfaces that lack abundant moisture and nutrient availability, most microorganisms that arrive from other environments (such as from human occupants) are generally considered unlikely to survive, and those viable microbes that do survive are generally considered to be inactive or dormant until transferred to other host locations or until they experience an influx of moisture and nutrients that help them proliferate [4••, 50–52]. Surveys of fungal communities in indoor environments, conducted using high-throughput molecular sequencing, have shown that they tend to be driven primarily by transport from the local outdoor environment [31]. However, similar surveys of bacterial communities in the built environment have revealed high abundances of skin-associated bacteria (e.g., *Propionibacterium acnes*, *Corynebacterium*, and *Streptococcus*), particularly in buildings and on surfaces with high human occupancy and frequency of interactions [35]. Source-tracking efforts have also provided insight into the origin of the bacteria that reside on various indoor surfaces. For example, urine- and feces-associated bacteria have been shown to be more common on toilet seats and toilet handles than on other surfaces [53]; bacteria associated with fresh produce have been shown to be more common on kitchen countertops and inside refrigerators [54]; and bacteria associated with leaves and soil have been shown to be more common on the interior and exterior trim of doors that open to the outside than other (more interior) home surface locations [55]. Conversely, on surfaces that frequently have high moisture levels, such as those in bathrooms and kitchens (e.g., shower curtains, sinks, and countertops), rich microbial biofilms can form community assemblages that closely resemble those found in plumbing systems and water reservoirs [56–58]. Investigating differences both within and between homes, Lax et al. (2014) demonstrated that bacterial communities on different surfaces in an individual home showed strong similarities for some surfaces (e.g., kitchen floors were similar to bedroom floors and both were similar to human feet; and kitchen light switches were similar to the front doorknob, which were also similar to occupants’ hands) but not for others (e.g., kitchen countertops and human noses were distinct from doorknobs) [3••]. Moreover, when a family moved homes, the bacterial community composition on surfaces in the new home rapidly converged toward the composition of bacteria from surfaces in the previous home, suggesting that the new occupants quickly deposited their own unique signatures of human-associated bacteria to the new space.

While much has been revealed about bacterial and fungal communities in indoor environments in recent years, much less is known about viral communities and total viral abundance on surfaces in buildings [59]. However, much has been learned about the presence, abundance, and survival of specific viruses and other potential pathogens that cause concern for infectious disease transmission and other emerging microbial hazards.

### Viral Pathogens on Fomite Surfaces

#### Presence and Abundance

The study of fomites has traditionally involved determining whether the presence of specific potentially pathogenic organisms—primarily those of viruses or bacteria—resided on environmental surfaces. For example, in an early influential survey, Boone and Gerba (1982) sampled over 300 fomites from daycare centers and homes to determine the presence of influenza A virus on each surface [60]. During flu seasons, approximately half of all common building surfaces from both types of indoor environments had measurable levels of influenza virus, suggesting that contaminated fomite surfaces could play a role in influenza transmission.

Since then, numerous similar studies targeting influenza and other viruses have also discovered that, for example:

- Norovirus and influenza A virus were found on frequently used fomites (e.g., desktops, faucet handles, and paper towel dispensers) in elementary school classrooms [61].
- Widespread norovirus contamination was found on fomite surfaces on houseboats on which an outbreak of norovirus gastroenteritis was suspected [62].
- Picornavirus (including rhinovirus and/or enterovirus) was detected on approximately 20% of toys in pediatric office waiting rooms [63].
- Human rhinovirus (hRV) was detected on 5% of clothing samples from teachers working in childcare centers [64].
- Rotavirus was detected on about 20% of fomite samples in daycare centers, including on telephone receivers, drinking fountains, water-play tables, and toilet handles [65]; and on nearly half of surfaces sampled in a pediatric unit, with higher prevalence on surfaces that are commonly in direct contact with children (e.g., thermometers and play mats) than on other environmental surfaces (e.g., door handles and wash basins) [66].
- Severe acute respiratory syndrome (SARS) coronavirus RNA was found on 30% of surface swab samples in hospitals, including in patient rooms, on computer mice at nurse stations, and on the handrail of a public elevator [67].
- Human parainfluenza virus 1 (HPIV_1_) was detected on 37% of a total of 328 fomites from 12 different office buildings, most frequently isolated on desktops [68].
- HPIV_3_, HPIV_1_, and norovirus GII RNA were detected on 16 (12%), 7 (5%), and 4 (3%) of a total of 130 surfaces sampled in offices, with computer keyboards, computer mice, telephones, and desktops having significantly higher abundances than other fomite surfaces such as door handles, light switches, or ventilation ducts [5•].
- Human adenoviruses (HAdV) were detected from 63 of 141 (45%) fomite samples in an adult intensive care unit (ICU) in a hospital in Rio de Janeiro, Brazil, with viral loads ranging from 2.48 × 10^1^ to 2.1 × 10^3^ genomic copies per milliliter [69].
- Middle East respiratory syndrome coronavirus (MERS-CoV) was detected on 2 of 51 (4%) high-touch surfaces in patient rooms with laboratory-confirmed MERS-CoV patients [70].

These studies and many others confirm that viruses that are known to cause communicable diseases in humans are commonly found on surfaces, but it then must be determined whether they are viable and potentially infectious to humans.

#### Viability and Survival

Weber and Stilianakis (2008) reviewed numerous studies that investigated the environmental inactivation of influenza A viruses, finding that daily inactivation rate constants differ by several orders of magnitude depending on the nature of surface characteristics and that influenza virus can survive in aerosols for several hours, but only for a few minutes on human hands [71]. As an example from this body of literature, Bean et al. (1982) tracked the survival of laboratory-grown influenza A and B viruses on various surfaces, finding that both viruses survived up to 48 h on hard, non-porous surfaces, such as stainless steel and plastic and up to 12 h on porous surfaces, such as cloth, paper, and tissues [72]. Moreover, fomite transmission of influenza viruses was considered possible because influenza virus could be transferred from stainless steel surfaces to hands for up to 24 h after deposition (and from tissues to hands for up to 15 min after deposition). The viruses then subsequently survived on hands for an additional 5 min after transfer from the tested fomites.

More recently, Greatorex et al. (2011) combined the two main types of approaches commonly used in the literature to evaluate the survival of influenza A and pandemic H1N1 viruses inoculated onto a wide range of surfaces common to work and home environments [73]: (i) molecular (genomic) detection by reverse transcription polymerase chain reaction (RT-PCR), which provides a quantitative measure of presence/abundance of genetic material, and (ii) virus viability by plaque assay (for influenza A) or fluorescent focus assay (for H1N1), which provides a measure of virus survivability. The genome of both viruses was detected on most surfaces up to 24 h after inoculation with minimal decrease in gene copy number (except for unsealed wood surfaces), while virus viability decreased more rapidly to a level below detection on all surfaces at 24 h. However, viruses did survive up to 4 h on most surfaces and up to 9 h on non-porous surfaces. The authors concluded that influenza A transmission via fomites is possible, but it is unlikely if contact occurs after long periods following surface contamination, unless re-inoculation occurs during that time. Similarly, Mukherjee et al. (2012) investigated the viability of H1N1 virus on naturally contaminated hands and household surfaces of 20 individuals with laboratory-confirmed infection, finding that H1N1 has a short period of survival on naturally contaminated skin and fomites, and secretions deposited on hands by coughing or sneezing have a concentration of approximately 20–30 TCID_50_/mL [74].

Others have found that influenza virus can survive (i.e., remain viable and/or potentially infectious) much longer on fomite surfaces, using a variety of approaches. Thomas et al. (2008) tested the survival of influenza A viruses on banknotes after intentional contamination, finding viruses could survive up to 3 days after inoculation at high concentrations [75]. Additionally, when the virus was encapsulated in respiratory mucus (which may more realistically reflect human contributes to fomite surfaces), survival was as high as 17 days. And when nasopharyngeal secretions from naturally infected children were used to inoculate banknote surfaces, influenza virus survived at least 2 days in one-third of the test cases. Similarly, Oxford et al. (2014) found that influenza A H1N1sw virus particles survived and remained infectious for up to 48 h on a wooden surface, for 24 h on stainless steel and plastic surfaces, and for 8 h on a cloth surface [76]. Perry et al. (2016) found that two influenza A (H1N1) virus strains deposited on stainless steel surfaces remained infectious over a weeklong period, with a 2-log_10_ loss (99%) in infectivity over 7 days [77]. Moreover, infectivity decreased more rapidly over time at higher absolute humidity, which is consistent with other similar studies [78, 79]. Thompson et al. (2017) tested the viability and RNA abundance (via qt-RT-PCR signal) of five influenza strains seeded on three surfaces (cotton, microfiber, and stainless steel) over time, finding that viable virus was detected for up to 2 weeks on stainless steel and up to 1 week on cotton and microfiber samples [6•]. Times to achieve 99% reductions in viability were ~ 18 h for cotton, ~ 34 h for microfiber, and ~ 175 h for stainless steel. Specific to materials used in personal protective equipment (PPE), Sakaguchi et al. (2010) found that the infectivity of influenza A virus was maintained for ~ 8 h on the surface of an N95 particulate respirator, a non-woven fabric surgical mask, a Tyvek gown, a coated wooden desk, and stainless steel, and for ~ 24 h on a rubber glove [80], suggesting that frequent replacement of PPE and clothing worn by healthcare professionals is warranted to minimize cross-infection. While there is high variability among these studies in influenza inactivation rates and survival on fomite surfaces over time (influenced by a combination of material type, environmental conditions, virus strain, inoculation methods, and viral presence/abundance/viability detection methods), there is general consistency in the literature that influenza viruses can survive for up to several days after being deposited on some surface types and in some conditions.

The survival of other viruses on fomites has also been investigated in recent years. For example, in the aforementioned study of HAdV in an ICU unit in Brazil, a subset of 10 samples that were positive for HAdV were selected for viability assessment, and exactly half of those samples were indeed still viable [69]. Boone and Gerba (2007) reviewed prior studies of the viability of numerous respiratory and enteric viruses on surfaces, reporting virus inactivation rates ranging from ~ 0.01–0.1 log_10_ per hour for avian influenza and influenza A and B to ~ 0.2–0.6 log_10_ per hour for rhinovirus 14, PIV_2_, and respiratory syncytial virus [81]. Inactivation rates for enteric viruses were lower, from ~ 0.002–0.003 log_10_ per hour for astrovirus (serotype 4) and rotavirus p13 to ~ 0.01 log_10_ per hour for adenovirus 40. van Doremalen et al. (2013) reported that MERS-CoV viability was more stable at low temperature and low humidity conditions and could still be recovered after 48 h, suggesting fomite transmission of MERS-CoV is possible [82].

### Bacterial Hazards on Fomite Surfaces

In addition to viruses, bacterial hazards have also been found on fomite surfaces, including potentially pathogenic and antibiotic-resistant bacteria, which are often not mutually exclusive.

#### Potentially Pathogenic Bacteria

Marks et al. (2014) detected viable *Streptococcus pyogenes* and *Streptococcus pneumoniae* in samples from a daycare and then verified in laboratory tests that isolates of both organisms remained viable over extended periods of time and remained infectious in a mouse model when present as a biofilm (rather than as desiccated cells on surfaces) [83]. These findings suggest that fomite transmission in the environment could be an important pathway if fomites are contaminated with oropharyngeal secretions containing biofilm streptococci. Jones and Lutz (2014) measured the mean survival time of *Pseudomonas aeruginosa* on laminate, glass, and stainless steel surfaces to be 3.75, 5.75, and 6.75 h, respectively [84]. Malcolm et al. (2017) evaluated the growth and survival of the non-tuberculous mycobacterium (NTM) *Mycobacterium abscessus* in the presence of mineral particles, kaolin, halloysite, silicon dioxide, and house dust. *Mycobacterium abscessus* interacted with the particulates, with increased survival rates in the presence of house dust, surviving desiccation for as long as 2 weeks [7•]. These studies and others confirm that potentially pathogenic bacteria are present in the built environment and that they can survive on fomites for long periods of time.

#### Antibiotic-Resistant Bacteria

Antibiotic-resistant bacteria have been studied in even more detail than potentially pathogenic bacteria. In 2013, the US Centers for Disease Control and Prevention (CDC) published an analysis of the major antibiotic-resistant threats in the USA [85]. Davis et al. (2012) reviewed published works about the household transmission of *Staphylococcus aureus* and other staphylococci, and suggested that household microbial communities might have a role in the transfer of antimicrobial resistance genes and could be reservoirs for recolonization of humans [86]. Public transit environments can also play an important role, as handrails of public buses [87], as well as the hands of bus riders [88], in two cities in Portugal were tested positive for contamination by methicillin-resistant *Staphylococcus aureus* (MRSA).

Some of the greatest concerns for antibiotic-resistant bacteria transmission occur in healthcare environments where contamination and transmission are possible through numerous fomites, ranging from mobile phones [89] to medical devices [90] to surgical tape [91] to doctors’ handbags [92]. While it has been hypothesized that many of these fomites have been important sources, closer investigation often reveals a more nuanced understanding. For example, Julian et al. (2011) sampled the surfaces of cellular phones carried by personnel at a veterinary hospital for both MRSA and methicillin-resistant *Staphylococcus pseudintermedius* (MRSP). MRSP was isolated from only 2 of 123 phones, and MRSA was isolated from only 1 of 123 phones [93]. Similarly, Missri et al. (2018) assessed bacterial colonization on healthcare workers’ mobile phones in a hospital that were sampled immediately before and 5 min after sanitization with bactericidal wipes [8•]. All phones were colonized with bacteria, and healthcare workers had higher bacterial colonization than administrative staff. However, potential pathogens were detected on approximately one-third of phones (most commonly by *Staphylococcus aureus*), while only one phone was colonized with MRSA. No multi-drug resistant bacteria were detected. Smibert et al. (2018) swabbed medical staff personal mobile phones, departmental phones, and ICU keyboards and cultured for 94 multi-drug resistant organisms (MRDOs) that had been previously cultured from ICU patients, including 11 MRSA, 2 VRE, and 81 Gram-negative bacteria [9••]. MRSA was isolated from only two phones, and whole-genome sequencing of mobile phone isolates demonstrated the isolates on mobile phones had different single nucleotide polymorphism (SNPs) compared with the clinical isolates, which suggests that these fomites are unlikely to contribute to hospital-acquired MRDOs. Given the ubiquitous nature of bacteria in the built environment, studies that have characterized bacterial colonization alone tend to be less useful for yielding mechanistic or health-relevant insights than those that have targeted specific pathogens and other microbial hazards.

In addition to MRSA, other major microbial hazards in healthcare environments include *Clostridioides difficile* (*C. diff*), carbapenem-resistant Enterobacteriaceae, vancomycin-resistant *Enterococcus* (VRE), and a number of single- and multi-drug-resistant organisms [85]. Haun et al. (2016) reviewed 72 studies that assessed contamination of fomites in healthcare settings and found high variability in contamination rates by fomite type, by microbial agent (including MRSA, Gram-negative rods, enterococci, and *C. diff*), and by microbiological sampling and analysis technique [10•]. Grimmond et al. (2018) sampled for *C. diff* on 50 disposable and 50 reusable sharps containers in seven hospitals, finding that 8% and 16% of containers had detectable, albeit sub-infective, levels of *C. diff*, suggesting that sharps containers are not likely to pose a risk of *C. diff* transmission [94]. Jackson et al. (2019) sampled the bacterial burden on body sites of ICU patients who were colonized with vancomycin-resistant *Enterococcus* (VRE) and the healthcare workers (HCWs) who tended to those patients [11••]. HCW contamination on gloves and gowns (i.e., personal protective equipment or PPE) was associated with the VRE burden on body sites of patients with VRE, including perianal, stool, and skin swab samples, suggesting that ICU patients with a higher bacterial burden were more likely to transmit VREs to HCWs via their PPE.

A number of methods for controlling antibiotic-resistant bacteria and other microbial hazards on fomite surfaces have been investigated, including UV light, disinfectant cleaners, material coatings, and others. For example, Mitchell et al. (2019) quantified the doses of UV light that are required to inactivate MRSA, VRE, *C. diff*, and murine norovirus on stainless steel and Formica laminate fomite surfaces [95]. Reitzel et al. (2014) characterized the ability of a novel chlorhexidine and gentian violet antiseptic coating to kill bacterial and fungal pathogens on the surface of disposable medical gloves, finding that the coating eradicated MRSA, VRE, and multi-drug-resistant *Pseudomonas aeruginosa*, among others [96]. Despite the effectiveness of antimicrobial cleaners, other studies suggest that caution should be practiced in their use. For example, Hartmann et al. (2016) identified antibiotic resistance genes (ARGs) in settled dust from athletic and educational facilities, and found that ARG abundance was positively correlated with the concentration of antimicrobial chemicals found in the same dust samples [12••]. Similarly, Mahnert et al. (2019) compared the microbial communities and their resistomes (the total antibiotic resistance gene profile of a community) on surfaces of clinical settings using metagenomic genome and plasmid reconstruction, where they found that the microbiome of highly maintained built environments has a different resistome compared with other built environments, as well as a greater diversity of resistance genes [13••]. How these results are best applied is still an active area of research, as ARGs are also natural components of environments rich with bacteria (e.g., soils), and their role in shaping bacteria in indoor environments is not yet well understood.

One promising area of research that has emerged in recent years may offer an alternative to traditional cleaning methods. Unlike antimicrobials that kill microbes, probiotic cleaners that contain spores from *Bacillus* species (i.e., *B. subtilis*, *B. pumilus*, and *B. megaterium*) are thought to work primarily through biological competition to prevent the survival and proliferation of pathogenic bacteria [97, 98]. Probiotic cleaners have been found to be more effective than traditional cleaning methods, with several studies demonstrating that their use decreased pathogen load on surfaces by an average of 90% more than conventional chemical cleaners (ranging from 70 to 99%; [99, 100]). Furthermore, Caselli et al. (2019) showed that in hospitals where probiotic cleaners were used, the abundance of antibiotic resistance genes on treated surfaces was reduced by up to 99% [101]. Importantly, Caselli et al. (2016) confirmed their safety for use in healthcare facilities by measuring the infection rate from over 30,000 patients across seven facilities and found no evidence of infection by *Bacillus* spp., regardless of whether patients were at high risk for infection by opportunistic pathogens [102]. Safe sterilization or eradication of antibiotic-resistant bacteria on fomite surfaces remains an active area of study.

The aforementioned studies confirm that not only do pathogenic viruses, non-pathogenic viruses, and bacteria deposit and exist on fomites in the built environment they can also remain viable for hours, or even days, dependent upon the fomite material, microorganism type, and indoor environmental characteristics. From there, are they transmitted to humans and, if so, what are the implications of fomites for human health?

## Transmission Between Fomites and Humans

**Fig. 1:**
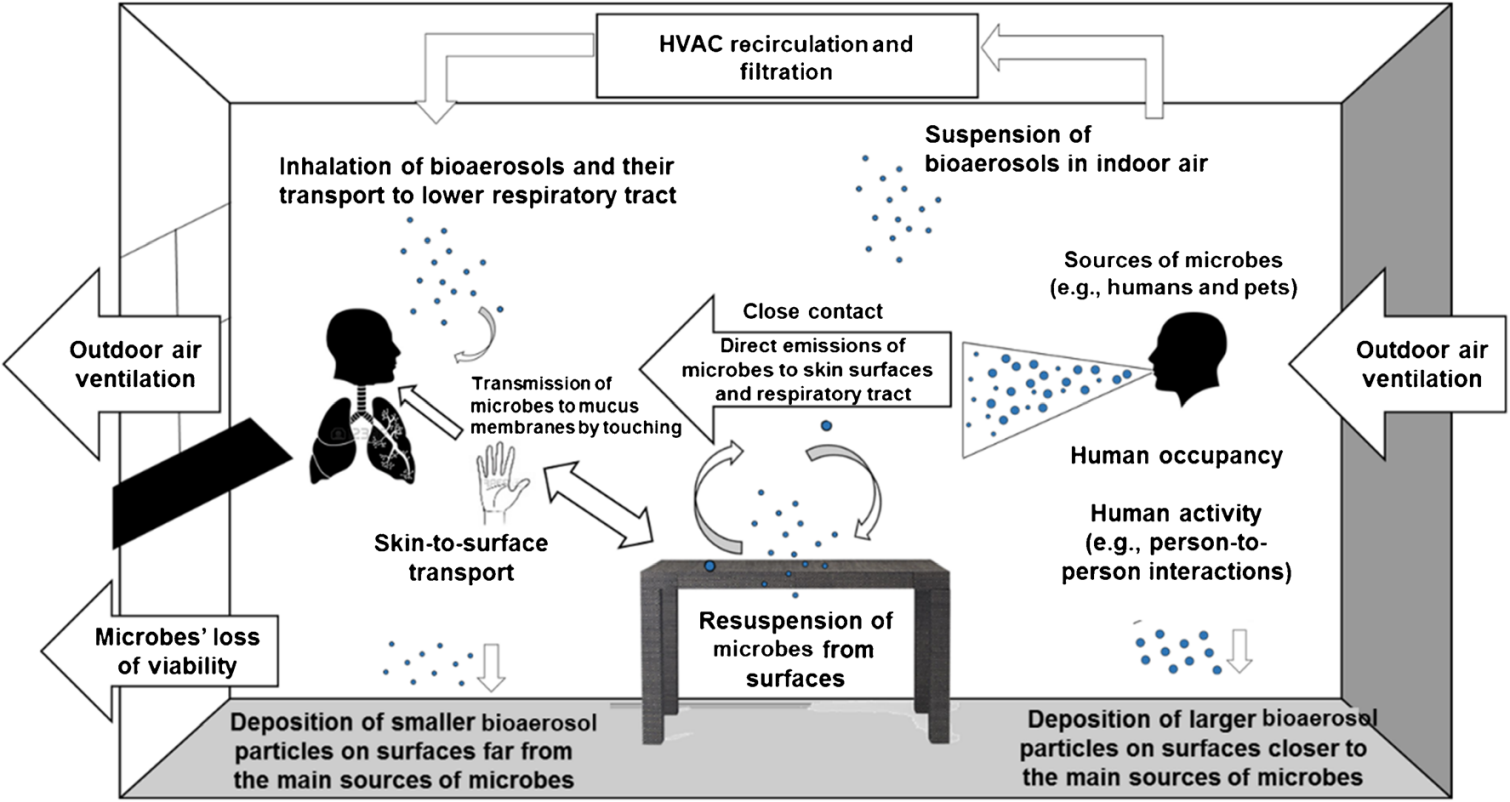
Conceptual figure demonstrating various microbial transmission pathways between humans, air, and fomites in a typical indoor environment

In addition to detecting genetic material and viable microbes on numerous fomite surfaces, it is also crucial to understand the factors that affect the likelihood of transmission between fomites and humans. Three key approaches have been used to provide insight on the importance of fomites and other potential modes of transmission for various microbial hazards and their impacts on human health: (i) experimental measurements of the transfer of microbes to/from fomites and humans, (ii) mathematical modeling of microbial exchange between fomites and humans and subsequent health risks in the context of other exposure pathways (e.g., direct contact and aerosol exposure), and (iii) epidemiological studies designed to elucidate the importance of different modes of transmission in causing disease. Figure 1 illustrates routes of microbial transfer to and from fomites, air, and humans in a typical indoor environment.

### Measurements of Microbial Transfer to/from Fomites

Numerous studies have experimentally characterized the transfer of microbes to and from fomites and humans in the built environment, including those that have focused on dynamics at the surface-scale and room-scale.

#### Surface-Scale

In one surface-level transfer dynamics study, Tuladhar et al. (2013) measured the transfer of human norovirus (NoV) between fingers and fomites, as well as between fingers and food products. They artificially contaminated human finger pads and pressed them on laminate surfaces, stainless steel surfaces, whole tomatoes, and cucumber slices. In addition, they contaminated the surfaces themselves and pressed clean human finger pads against those same surfaces [103]. Initial transfer efficiencies on the first pressing averaged ~ 13%, decreasing over time and after drying of the contaminated finger pads. The transfer efficiency for a viable, infectious virus from surfaces to finger pads was between 2 and 4%, on average, even after 40 min of drying the contaminated surfaces. A number of other surface-scale dynamic studies have focused on the transfer efficiency between different types of organisms and different types of fomites that are common in healthcare settings, such as medical gloves. For example, Moore et al. (2013) evaluated MRSA transmission between different types of gloves worn by HCWs and fomite surfaces, finding that bacterial transfer ranged from ~ 0 to ~ 20% and varied depending on glove material and material hydrophobicity, while the adsorption of simulated body fluids increased bacterial transfer and also made transfer more uniform across glove types [104]. Greene et al. (2015) estimated the transfer efficiency of *Acinetobacter baumannii*—a drug-resistant healthcare-associated pathogen—with and without latex glove use from the finger pad to a fomite and from a fomite to the finger pad, testing six materials (i.e., glass, stainless steel, porcelain, polypropylene, polycarbonate, and rubber) [14•]. Without gloves, the fomite-to-finger-pad transfer efficiency was ~ 24% and the finger-pad-to-fomite transfer efficiency was ~ 6%. Latex gloves reduced both of these transfer efficiencies by about half, and material type was not a major determining factor. Koenig et al. (2016) measured the transfer efficiency of *Staphylococcus aureus* between nitrile exam gloves and non-porous fomites via handshaking with another person with gloved hands, touching a plastic cellular phone back, and touching a stainless steel rod [105]. The highest transfer efficiency was with the steel rod, followed by the cellular phone back. Glove-to-glove transfer occurred but had the lowest transfer efficiency among the three scenarios studied. Lopez et al. (2014) quantified fomite-to-finger microbial transfer of *Escherichia coli*, *Staphylococcus aureus*, *Bacillus thuringiensis* spores, and poliovirus 1 seeded on ceramic tile, laminate, and granite after treatment with a disinfectant wipe; microbial transfer was much lower on treated fomites than non-treated fomites (i.e., up to < 0.1% vs. up to 36%, on average) [106]. These studies and others provide helpful quantitative information about the physical microbial exchange to/from fomites and humans, which provides helpful context for fomite transmission and also informs mathematical models of microbial exchange in the built environment.

#### Room-Scale

Room-level dynamics studies have used a number of experimental approaches to elucidate the importance of fomite transmission to and from humans. For example, Winther et al. (2007) assessed rhinovirus contamination of environmental surfaces by housing 15 adults with naturally acquired rhinovirus colds in a hotel overnight and having them conduct a combination of natural and scripted activities [107]. Thirty-five percent (35%) of 150 environmental sites sampled in the rooms were contaminated with rhinovirus. Moreover, rhinovirus was successfully transferred from surfaces to fingertips in 60% of samples taken 1 h after scripted activities to intentionally transfer the virus from humans to surfaces, as well as in 33% of samples taken 18 h after the scripted activities. Killingley et al. (2016) quantified viral loads recovered from the nostrils of subjects infected with influenza A(H1N1)pdm09 and correlated those amounts with viral loads recovered from their immediate environment in community and hospital settings [15•]. The mean duration of virus shedding was ~ 6 days by PCR (molecular detection) and ~ 4 days by culture (viability detection). Only ~ 5% of surface swabs were PCR positive for influenza, and only 0.3% yielded viable virus; however, room air near a subset of the subjects was also sampled and was found PCR positive for influenza virus in ~ 40% of the samples, suggesting that the importance of aerosol influenza transmission is likely greater than indirect transmission via fomites. Suwantarat et al. (2017) combined microbial sampling with observations of hospitalized patients and reported that patients frequently had direct or indirect interactions with medical equipment and other fomites that are shared among patients, and that those items were often contaminated with health care–associated pathogens [108]. The surfaces that patients interacted with most frequently included medication carts, wheelchairs, food trays, and cleaning carts, resulting in between ~ 0.2 and ~ 1.4 interactions per hour.

Microbial tracers have also been used to investigate fomite transmission at the room-scale with some success. For example, Kunkel et al. (2017) used a human respiratory activity simulator to aerosolize two model organisms—*Escherichia coli* K12 and bacteriophage T4—in an unoccupied apartment unit operating with four different particle filters installed in the recirculating central forced air heating, ventilation, and air-conditioning (HVAC) system [16•]. Size-resolved aerosol sampling and settle plate swabbing were conducted in multiple locations, and samples were analyzed by DNA extraction and qPCR. DNA from both organisms was detected under all test conditions in all air samples up to 7 m away from the bioaerosol source, with concentrations decreasing at greater distances. A greater fraction of T4 DNA was recovered from the aerosol size fractions smaller than 1 μm than *E. coli* K12 at all air sampling locations, suggesting that smaller virus-like organisms can transport longer distances than the larger bacterial organisms. Moreover, higher efficiency particle filters in the HVAC system reduced the amount of DNA recovered in air samples, as well as on settle plates located 3–7 m from the source. In another microbial tracer study, Sassi et al. (2018) assessed the amount of surface contamination that occurs in restrooms during toilet flushing using coliphage MS2 added to the toilet bowl [109]. The toilet bowl rim, toilet seat top, and toilet seat underside were contaminated in all tests where no disinfectant was added to the bowl water before flushing, while the addition of disinfectant to the toilet bowl prior to flushing reduced concentrations on fomites after flushing. Similarly, Booth and Frost (2019) used a vomit simulator to investigate the distribution and survival of *Feline calicivirus* (FCV) as a surrogate for norovirus, demonstrating that viable virus was recovered from almost all samples taken from the floor up to 3 m away from the source, while no air samples contained viable virus [110]. In a highly novel tracer experiment, Reynolds et al. (2019) evaluated microbial transmission in an outpatient clinic and the impact of an ethanol-based disinfectant by placing a viral tracer (bacteriophage MS2) on two fomites at the beginning of the day: a patient room door handle and a front desk pen [17••]. Fomites and the hands of patients and staff were sampled after 2, 3.5, and 6 h. For the disinfectant intervention trials, high-touch surfaces were cleaned 4 h after seeding and sampled 2 h later. The viral tracer was detected on all surfaces and all hands sampled at all three time points, with examination room door handles and nurse station chair arms yielding the greatest concentrations. MS2 concentrations were greatest 2 h after inoculation, and virus concentrations decreased by ~ 94% after application of the disinfectant spray. If one can assume that microbial transfer efficiencies for MS2 are similar to those for other health-relevant organisms (which may not be the case [111]), then tracer studies like this can provide meaningful experimental evidence for the potential for fomite transmission in the built environment.

Combined, both surface-scale and room-scale studies clearly demonstrate that the role of fomites in the transmission of microbes to humans can be an important exposure pathway.

### Mathematical Modeling of Fomite Transmission

The combination of quantitative measures of (i) abundance of specific microorganisms on fomite surfaces, (ii) human contact frequency and interactions with fomites, and (iii) microbial transfer efficiency between humans and fomites also makes it possible to quantify the likelihood of microbial exchange and potential health risks using more detailed mathematical models of entire built spaces. These approaches are commonly referred to as quantitative microbial risk assessments (QMRA) [112].

#### Mechanistic Models

Detailed mechanistic models of disease transmission in the built environment combine (i) probabilistic fate and transport models to estimate the dose of potential pathogens delivered to infection sites of susceptible individuals and (ii) dose-response models to estimate the probability of infection based on estimates of the quantities of pathogens delivered to infection sites [113]. The underlying fate and transport models in these studies commonly use Markov chain models combined with single-zone or multi-zone mass balance models or computational fluid dynamics (CFD) models to simulate physical transport mechanisms, such as bioaerosol emissions, removal by ventilation, and deposition to fomite surfaces. A Markov chain is a random process that undergoes transitions from one state to another on a state space. Physical elements (e.g., room air, skin, and mucus membranes) and pathogen removal mechanisms (e.g., loss of viability, ventilation, and filtration) in the source environment-receptor pathways are represented as *states* in a discrete-time Markov chain model. Pathogens can be transferred and exchanged between states due to physical mechanisms. Markov chain models have been widely used for estimating doses of influenza virus in several environments, including healthcare facilities and airplanes [114–119].

As an early example of a Markov chain model application for estimating transmission routes of a respiratory pathogen, Nicas and Sun (2006) illustrated a hypothetical scenario in which a viral pathogen was emitted by a patient via coughing and transmitted to an attending HCW [115]. The model required detailed inputs on pathogen loads in coughs; deposition rates to and survival on surfaces; airborne inactivation and ventilation rates; rates of pathogen transfer to hands, mucous membranes, and respiratory tract; and dose-response model parameters. Their case study demonstrated the importance of the hand-to-mucous-membrane contact and droplet spray exposure routes for the case study and demonstrated model sensitivity to assumptions for each input parameter.

Other models have been used to simulate the transmission of specific pathogens. For example, Kraay et al. (2018) developed a compartmental model that accounts for fomite transmission of viral pathogens, including contacting fomites after shedding onto those surfaces and shedding onto hands. This model then predicted influenza, rhinovirus, and norovirus transmission in a daycare, subway, office, and school [120]. They predicted that fomite transmission for rhinovirus and norovirus can sustain transmission in all locations, while fomite transmission is likely not sustained for influenza. Xiao et al. (2017) used a multi-agent model to predict the distributions of infection risk during the well-known Ward 8A SARS outbreak in 2003 in the Prince of Wales Hospital in Hong Kong, concluding that the SARS coronavirus was most likely spread via a combination of long-range airborne and fomite routes [18•]. Sze-To et al. (2013) modeled the impacts of surface material, ventilation rates, and human behavior (e.g., close contact rates between individuals) on the transmission of influenza A, respiratory syncytial virus (RSV), and rhinovirus in a hospital ward and aircraft cabin. They predicted that a reduction in close contact rates is more effective than an increase in ventilation rates at decreasing infection risk, and fabric surfaces present a much lower risk of transmission than non-fabric surfaces [121]. Similar results have been obtained for SARS and MERS in other studies as well [122]. For example, Lei et al. (2018) modeled in-flight outbreaks of influenza A H1N1, SARS, and norovirus in an air cabin, and predicted that the dominant route of transmission was a close contact for influenza and contact with fomites for SARS and norovirus [123]. Many others have also developed and applied models for investigating the transmission dynamics of generic [124] and specific pathogens, including influenza [125–128], norovirus [129], rotavirus [130], and cholera [127].

#### Improving Model Inputs

A number of recent studies have also focused on improving the accuracy of model inputs to increase the overall accuracy and relevance of mechanistic transmission models. For example, since models have shown that human behaviors (e.g., close human-to-human and human-to-fomite contact rates) are major drivers of variability in infection risk models, several studies have used videography and visual observations to characterize and quantify these inputs. Nicas and Best (2008) videotaped 10 subjects for 3 h while performing office-type work in isolation from others, recording the number of contacts to the eyes, nostrils, and lips [131]. Julian and Pickering (2015) combined videography with a microbial sampling of fomite surfaces to develop fecal indicator bacterial exposure profiles at very high time resolution (i.e., 1-s intervals) in Tanzania [132]. Zhang and Li (2018) characterized more than 3500 person-to-person contacts and 127,000 surface touches in a student office space obtained by video camera. They then used those data as model inputs to predict the intranasal dose of influenza A viruses to students and surrounding fomites and to evaluate the effectiveness of various control strategies [19•]. Hertzberg et al. (2018) chronicled the behaviors and movements of individuals in the economy cabin on single-aisle aircraft during 10 transcontinental US flights to provide information on the movements of passengers and crew that may facilitate disease transmission [133]. They simulated droplet-mediated transmission, predicting that there is a low probability of direct transmission to passengers not seated in close proximity to an infectious passenger. They also collected 229 air and surface samples during flights, and although eight flights were during the flu season, all qPCR assays for 18 common respiratory viruses were negative. Smieszek et al. (2019) used wireless sensors to measure the location and close proximity contacts among individuals at a high school in the USA, which allowed for modeling droplet and aerosol transmission of influenza both in isolation and in combination [134]. Importantly, Greene et al. (2018) used a model of *Acinetobacter baumannii* to demonstrate how incorrect assumptions for pathogen transfer efficiency between fomites and fingers (and between fingers and fomites) can adversely impact model predicted results [20•].

Another key parameter that affects not only model results but also the underlying mechanistic studies of surface interactions is sample recovery efficiency (SRE). Herzog et al. (2012) demonstrated that the SRE of bacteriophage P22 applied to a number of different fomites under a variety of conditions was most influenced by sampling time, fomite surface area, wetting agent use, and relative humidity [135]. Ganime et al. (2015) evaluated swab sampling as a method to recover murine norovirus 1 (MNV-1) and bacteriophage PP7 from porous, non-porous, and rubberized fomite surfaces, finding a highly variable recovery efficiency ranging from < 1 to 77% [136]. Weir et al. (2016) demonstrated the impact that variability in fomite recovery from surfaces using different sampling methods can have on QMRA model results [21•]. They also reported measurements of the recovery efficiency for enterobacteria phage P22 using combinations of different sampling tools (i.e., swabs and wipes) and eluents (i.e., polysorbate 80, trypticase soy broth, and beef extract) on different non-porous fomites (i.e., aluminum, ceramic, glass, plastic, steel, and wood laminate), finding that polysorbate 80 wipes with a surface area of 10–100 cm^2^ had the highest sample recovery efficiency. As models continue to be developed, refined, and applied, the importance of having accurate input parameters continues to grow, as it has become increasingly clear that they are essential in the development of realistic and useful models.

### Epidemiology of Fomite Transmission

Epidemiological investigations offer the benefit of increasing our understanding of overall disease transmission and attack rates in exposed populations, but they are often limited in their ability to disentangle infectious disease transmission through various exposure routes. In large observational cohort studies, conducting exposure assessments that parse out the various modes of transmission is impractical. As such, researchers often rely on so-called natural experiments, like an outbreak in transportation environments, to make inferences about exposure pathways. For example, in a norovirus outbreak on an airplane, flight crews across several subsequent shifts became ill up to 5 days after an infectious passenger vomited in the airplane, confirming research on fomite transmission of norovirus [137]. For other viruses, like influenza, where fomite transmission is less well understood, natural experiments offer less clarity. For example, in another study of an airplane disease outbreak, 54 passengers aboard an airplane that experienced a 3-h ground delay with no ventilation and one infectious person, 72% of passengers developed symptoms [138].

As an example of an indirect human epidemiological study, Knox et al. (2012) investigated intra-household *S. aureus* transmission using a sample of multiple member households in New York City, NY [139]. Household members and standardized household environmental fomites were swabbed and cultured for *S. aureus*. *Staphylococcus aureus* colonized individuals in 62% of households and contaminated the environment in 54% of households. Environmental contamination was associated with transmission among households. Somewhat similarly, Cowling et al. (2013) applied a mathematical model to empirical data on influenza A transmission from randomized controlled trials of hand hygiene and surgical face masks in Hong Kong and Bangkok households [140]. They used inferences on the importance of close-range and long-range transmission modes, including information on the timing of secondary infections and apparent differences in the clinical presentation of secondary infections resulting from aerosol transmission. They estimated that long-range aerosol transmission via small particles (i.e., droplet nuclei) accounted for approximately half of all influenza transmission events. Lee and Wong (2015) conducted an epidemiological analysis of MERS-CoV transmission in South Korea and concluded that fomite transmission might have explained a significant proportion of the infections that occurred in the absence of direct contact with infected cases [141]. Despite some of these useful approaches, Kutter et al. (2018) recently summarized the state of knowledge of dominant transmission routes for a number of human respiratory viruses and noted that many studies on inter-human transmission routes remain inconclusive [22•].

Intervention studies, while rare and difficult to conduct, provide the best opportunity to empirically isolate transmission pathways. In an early study on this subject, Dick et al. (1987) experimentally investigated the plausible routes of transmission of rhinovirus colds by infecting healthy male adults with rhinovirus type 16, waiting for the onset of symptoms, and then having the infected individuals play cards with susceptible male adults in a room for 12 h [142]. Some of the recipients were allowed to function normally, retaining the ability to touch the cards and their faces, meaning that infection could plausibly occur via a combination of aerosol, direct contact, and/or indirect fomite contact. Meanwhile, other recipients were restrained with a harness, such that they could not touch their faces, thereby eliminating the possibility of fomite transmission. Strikingly, there was no statistically significant difference in infection rates between the two groups, suggesting that aerosol transmission was the dominant mode of transmission among individuals. Moreover, another experiment reported in the same study attempted to spread rhinovirus via heavily contaminated fomites alone, and no infections occurred among the recipients. This highly novel study, with an approach that has not been repeated for other organisms or other settings to date, provided unique epidemiological evidence of the importance of aerosol transmission—and the lack of importance of fomite transmission—for rhinovirus colds in humans.

## Conclusions

This review summarizes our understanding of fomite contamination, microbial survival, microbial exchange, and associated human health risks in the built environment. Past efforts have relied on a combination of empirical measurements, mathematical models, and epidemiological approaches to yield novel insight into the magnitude of health risks to humans, the important modes of transmission, and therefore, effective strategies to use for controlling exposures. However, despite numerous recent advances, significant knowledge gaps remain regarding the dynamics of the transmission of infectious disease and other microbial hazards, the relative importance of long-range aerosol, short-range aerosol, direct contact, and fomites, and effective means for controlling exposures and reducing human health risks. Interdisciplinary research that integrates public health, microbiology, engineering, and architectural design is needed to address these knowledge gaps and to inform our current design standards and guidelines.
